# Supplementary material for: MetaRibo-Seq measures translation in microbiomes
Source: Nat Commun. 2020 Jun 29;11:3268. doi: 10.1038/s41467-020-17081-z (PMC7324362; doi:10.1038/s41467-020-17081-z)
Supplement: Supplementary file 10 — Supplementary Data 7 [file 41467_2020_17081_MOESM10_ESM.zip › File2/Confidence_High_Taxonomy/292759_out.krona.html]

Javascript must be enabled to view this page.

members
magnitude
magnitudeUnassigned
count
unassigned
taxon
rank

292759\_out

12

12
2
superkingdom

12
phylum
1239

186801
class
12

order
186802
12

186803
family
12


SRS014459\_contig\_number\_5252SRS015065\_contig\_number\_contig-100\_4936.4936SRS052697\_contig\_number\_contig-100\_15651.149672SRS148253\_contig\_number\_contig-100\_35142.35143SRS893358\_contig\_number\_contig-100\_5882.5883
5
12
33042
genus


SRS024132\_contig\_number\_47235SRS054956\_contig\_number\_contig-100\_1011.76601
2
1896996
species

410072
species

SRS020328\_contig\_number\_23564SRS050752\_contig\_number\_contig-100\_24169.64334SRS098644\_contig\_number\_contig-100\_18913.95341SRS1041091\_contig\_number\_19267SRS143523\_contig\_number\_contig-100\_4849.4850
5
